# SNP Linkage Analysis and Whole Exome Sequencing Identify a Novel *POU4F3* Mutation in Autosomal Dominant Late-Onset Nonsyndromic Hearing Loss (DFNA15)

**DOI:** 10.1371/journal.pone.0079063

**Published:** 2013-11-18

**Authors:** Hee-Jin Kim, Hong-Hee Won, Kyoung-Jin Park, Sung Hwa Hong, Chang-Seok Ki, Sang Sun Cho, Hanka Venselaar, Gert Vriend, Jong-Won Kim

**Affiliations:** 1 Department of Laboratory Medicine and Genetics, Samsung Medical Center, Sungkyunkwan University School of Medicine, Seoul, Korea; 2 Samsung Biomedical Research Institute, Samsung Medical Center, Seoul, Korea; 3 Department of Otorhinolaryngology-Head and Neck Surgery, Samsung Medical Center, Sungkyunkwan University School of Medicine, Seoul, Korea; 4 Centre for Molecular and Biomolecular Informatics, Radboud University Nijmegen Medical Centre, Nijmegen, The Netherlands; Yale School of Public Health, United States of America

## Abstract

Autosomal dominant non-syndromic hearing loss (AD-NSHL) is one of the most common genetic diseases in human and is well-known for the considerable genetic heterogeneity. In this study, we utilized whole exome sequencing (WES) and linkage analysis for direct genetic diagnosis in AD-NSHL. The Korean family had typical AD-NSHL running over 6 generations. Linkage analysis was performed by using genome-wide single nucleotide polymorphism (SNP) chip and pinpointed a genomic region on 5q31 with a significant linkage signal. Sequential filtering of variants obtained from WES, application of the linkage region, bioinformatic analyses, and Sanger sequencing validation identified a novel missense mutation Arg326Lys (c.977G>A) in the POU homeodomain of the *POU4F3* gene as the candidate disease-causing mutation in the family. *POU4F3* is a known disease gene causing AD-HSLH (DFNA15) described in 5 unrelated families until now each with a unique mutation. Arg326Lys was the first missense mutation affecting the 3^rd^ alpha helix of the POU homeodomain harboring a bipartite nuclear localization signal sequence. The phenotype findings in our family further supported previously noted intrafamilial and interfamilial variability of DFNA15. This study demonstrated that WES in combination with linkage analysis utilizing bi-allelic SNP markers successfully identified the disease locus and causative mutation in AD-NSHL.

## Introduction

Hearing loss is one of the most common hereditary diseases in human, and more than 70% of the cases are nonsyndromic (NSHL) [1], [2]. Most postlingual (late-onset) NSHL have an autosomal dominant pattern of inheritance (AD-NSHL), while prelingual (early-onset) NSHL is typically autosomal recessive (AR-NSHL) [3]. AD-NSHL represents one of the examples of extreme genetic heterogeneity in human with a growing list of causative genes particularly by virtue of the recent advances in genomics technology [4], [5]. A total of 27 genes from 64 loci (DFNA 1–64) and 41 genes from 95 loci and (DFNB1–95) have been identified for AD-NSHL and AR-NSHL, respectively (Hereditary Hearing Loss website; http://hereditaryhearingloss.org; last access in May 2013), which poses a big challenge to direct molecular genetic diagnosis of NSHL by conventional targeted gene analyses.

Linkage analysis is one of the powerful approaches for the identification of disease-causing genes, and single nucleotide polymorphisms (SNPs), typically bi-allelic markers, have advantages in its abundance in the human genome and ease of typing with a high throughput. Recent advances in sequencing technologies have enabled us to capture variants on a whole-exome or whole-genome scale. In particular, sequencing of the whole exome (whole exome sequencing [WES]) of selected individuals in an affected pedigree holds a promise for disease gene discovery in Mendelian disorders [6], [7]. However, the abundance of variants across genome or exome makes the selection of “disease-causing” mutations still challenging. Thus, narrowing down the genomic area in linkage with the disease significantly increases the power of WES in disease mutation identification.

In this study, we investigated on the deafness mutation in a large Korean family with AD-NSHL by a combination approach of linkage analysis using SNP chip and WES. As a result, we successfully identified a novel mutation in DFNA15 in the family.

## Materials and Methods

### Ethics Statement

Written informed consent was obtained from all participating individuals, and the study protocol was approved by the institutional review board at the Samsung Medical Center, Seoul, Korea.

### Proband and Audiometric Analyses

The proband (IV-20) was a 45-year-old Korean woman with NSHL since 21 years of age. Her family history revealed NSHL with an autosomal dominant inheritance. The molecular genetic test for the *GJB2* gene revealed no mutations. Audiometric analyses were performed in the proband and participating family members. In particular, pure tone audiograms (PTA) were obtained in each individual to determine hearing thresholds levels (dB) of air and bone conduction at frequencies ranging from 250 to 8,000 Hz on right and left ears independently, according to the standard protocols. Peripheral blood samples were collected from the proband and family members participating in the genetic diagnosis with given written informed consent.

### SNP Genotyping and Linkage Analysis

Genome-wide SNP genotyping was performed on DNA samples from 21 family members including the proband for linkage analysis (11 were affected and 10 were unaffected). The Illumina’s Linkage IV B Genetic Map v2 RevD panel was used for genome-wide SNP scanning (Illumina Inc. San Diego, CA, USA). This panel includes 6,008 SNP markers distributed evenly across the genome. The genetic map position of each SNP in the panel was determined by linear interpolation using NCBI build 35physical map position and a high-resolution STR genetic deCODE map. The mean and median intervals between markers were 488 Kb (0.62 cM) and 315 Kb (0.38 cM), respectively. Prior to genotyping, the yields of pure double strand genomic DNA samples were determined by using the Quant-iTTM PicoGreen® dsDNA Assay Kit (Eugene, Oregon, USA). Samples were then normalized to 50 ng/ul. Genotyping reactions were performed using Illumina Bead-Station kit reagents and protocols. The normalized genomic DNA (5 ul) from each sample was used as a template for Illumina Goldengate® Genotype Assays. SNP arrays were scanned using Illumina BeadArray Reader (Illumina Inc. San Diego, CA, USA) under the Illumina BeadStation 500G system version 2.3. For linkage analysis, we first excluded SNPs with a Mendelian inheritance error by using PedCheck 1.1 [8]. The Hardy-Weinberg equilibrium (HWE) test (*P*-value<0.01) was performed by using PEDSTATS 0.6.3 [9]. Linkage scores were calculated by MERLIN 1.1.2 [10]. Both muti-point parametric and nonparametric analyses were performed to yield LOD sores and NPL Z scores, respectively. In the parametric analysis, the disease allele frequency was set to 0.0001% and penetrance with 0, 1 and 2 copies of the disease allele was set to 0.01%, 100% and 100%, respectively. Haplotypes of the region of interest (peak linkage score) in the family were reconstructed by using PHASE 2.1.1 [11].

### Whole Exome Sequencing

**Figure 1:**
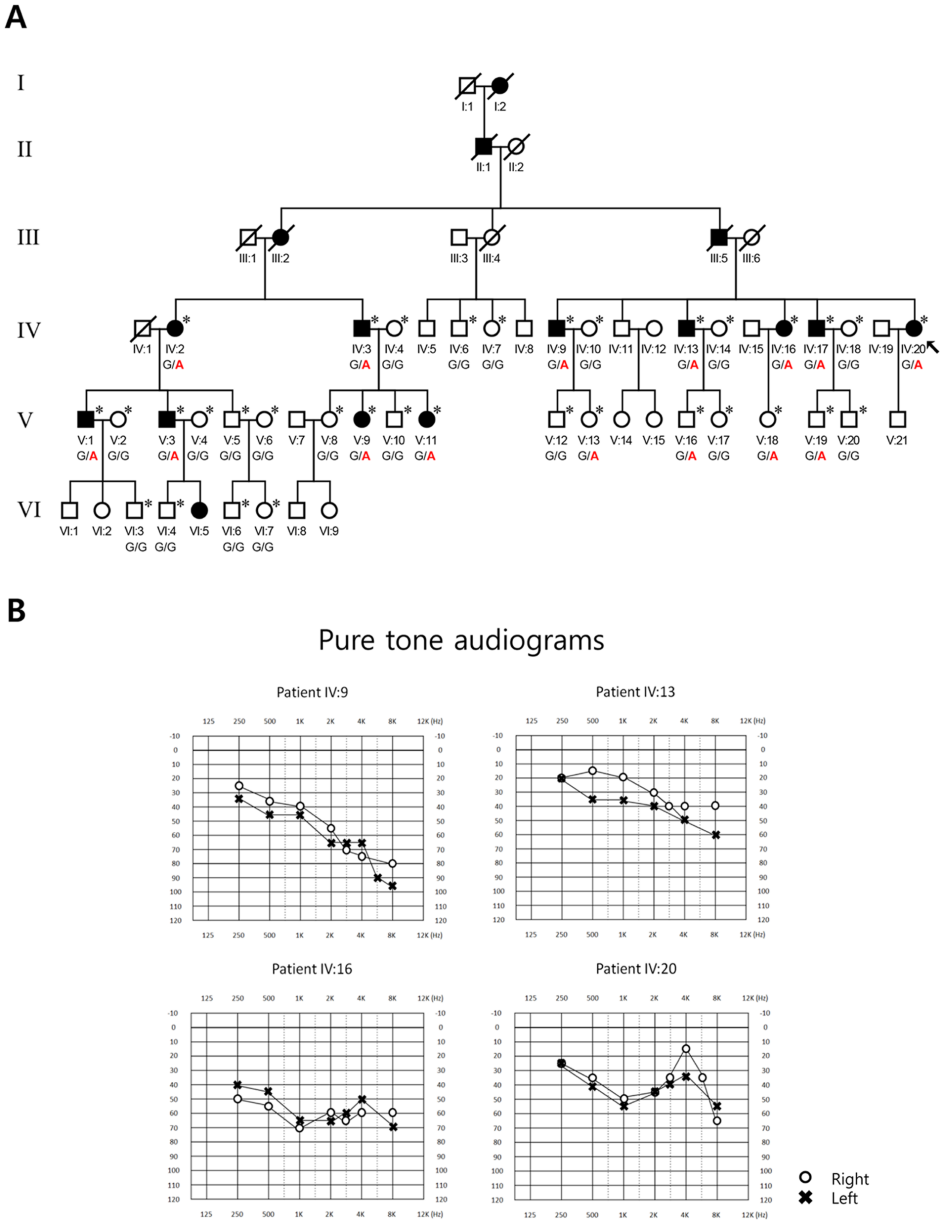
A large Korean family with autosomal dominant late-onset nonsyndromic hearing loss. (A) The pedigree with the proband (IV-20) indicated by an arrow. Individuals who participated in the molecular genetic diagnosis are marked with an asterisk on the right shoulder of symbols. Whole exome sequencing was performed in 4 individuals (IV:3, IV:17, V:1 and V:5). The genotypes at theArg326Lys mutation site of *POU4F3*are presented for each individual (mutant allele “A” in red). (B) Audiograms of 4 affected individuals in the family including the proband.

Four individuals in the family were selected for WES (3 affected including family member and 1 unaffected; IV:3, IV:17, V:1 and V:5 in the pedigree **Figure 1A**). The 64 million bases of exome were targeted by NimbleGen SeqCap EZ Human Exome Library v3.0 and sequenced by Illumina HiSeq. The 101-base-pair sequence reads were mapped to UCSC hg19 reference genome by using the Burrows-Wheeler Aligner [12]. Single nucleotide variants and insertion-deletion variants were detected by the SAMtools [13].

### Sanger Sequencing Validation and Bioinformatic Analyses

We performed Sanger sequencing for candidate variants from WES in 4 individuals (IV:3, IV:17, V:1 and V:5) and also in the remaining family members as indicated. Functional prediction, conservation and allele frequency in the 1000 Genomes Project and the Exome Sequencing Project 6500 data of missense variants were annotated using dbNSFP [14]. Functional effects of missense variants were predicted by five different programs, SIFT, PolyPhen-2, LRT, MutationTaster and MutationAssesor. In addition, sequence conservation of candidate variants was estimated by Genomic Evolutionary Rate Profiling++ (GERP) Rejected Substitution and conservation based on 29 mammals genomes. For molecular modeling, we used the structure of POU3F1 (2xsd) as a template to build a homology model for POU4F3 because the 3D-structure of POU4F3 is not yet experimentally solved. Modeling and subsequent analysis were done using an automatic script in the YASARA & WHAT IF Twinset with standard parameters [15], [16]. The effect of the mutation on the protein stability was estimated using the FoldX plugin in YASARA [16]. Control studies were performed by screening variants of interest involving DNA samples from 50 control individuals of Korean descent aged >60 years who had normal hearing by PTA.

## Results

### Proband and Pedigree

The detailed family history revealed a pedigree of a large Korean family (6 generations) with NSHL indicating the pattern of autosomal dominant inheritance (**Figure 1A** and **Table S1**). The age of onset of hearing loss in affected individuals ranged from early 10 s to late 50 s. No individual with hearing loss had syndromic features. PTA was obtained from 4 affected individuals including the proband (IV-9, IV-13, IV-16 and IV-20) in the family, which demonstrated bilateral minimal to moderate sensorineural hearing loss with flat to gently downward-sloping audiograms (**Figure 1B**). Peripheral blood samples were collected from total 34 individuals including the proband (indicated with an asterisk on the right shoulder of the symbols in **Figure 1A**).

### Linkage Analysis

Total 215 SNPs were excluded from linkage analyses due to Mendelian inheritance error. No markers failed the HWE test. The NPL analysis revealed 4 genomic loci with an NPL Z score >3. On parametric linkage analysis, we first obtained the LOD score without including 4 individuals in the generation VI (VI:3, VI:4, VI:6 and VI:7) whose affected status was ambiguous due to the young age. As a result, a high LOD score (2.387) was observed in the genomic region of 5q31, while the other 3 loci with NPL Z score >3 demonstrated no significant LOD score (**Table 1**). For those young 4 individuals (VI:3, VI:4, VI:6 and VI:7), we performed a simulation study by labeling them as unaffected or affected. The results showed that the LOD score was further increased to 3.006 when they were assigned as “unaffected”, while the score decreased to 2.408 when they were assigned as “unknown phenotype”. The LOD score was <1 (loss of linkage signal) when at least one individual among them was set as “affected”. The haplotypes reconstructed from 26 SNPs with an NPL Z score >3 revealed a single haplotype (#2) segregating with the hearing loss phenotype in the family (**Table S2)**, including individuals VI:3, VI:4, VI:6 and VI:7 whose haplotypes were assigned as “unaffected”.

**Table 1:** Chromosome regions with nonparametric linkage Z score (NPL Z)>3.0.

Chromosomeregion	Peak SNP	Position (cM)^a^	Position (Mb)	NPL Z	LOD^b^	Peak region (cM)	Peak region (Mb, hg18)
5p13∼p14	rs2034586	48.70	30.08	3.66	−8.99	16.38−60.61	6.12−38.07
5q31	rs1016344	136.60	134.60	3.66	3.01	135.86−152.10 (135.86−165.02)^c^	133.44−148.71 (133.44−160.43)^c^
10p15	rs713588	17.89	5.93	3.58	−9.27	4.44−32.09	1.96−13.10
12q22∼q23	rs1030048	108.27	96.16	3.73	−5.98	103.49−112.36	92.21−99.96

a Genetic distance from the p-terminus according to the deCODE high-resolution map.

b Scores obtained on simulation analyses yielding the maximum value on 5q31 (see text for detail).

c The peak region of 5q31 in parenthesis means the interval corresponding to the region with LOD >3.0.

### Whole Exome Sequencing and Mutation Identification

**Figure 2:**
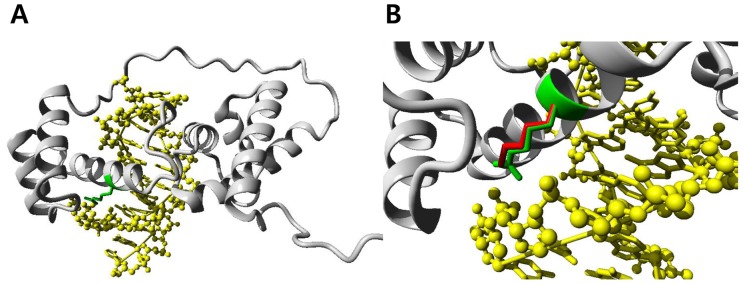
Molecular modeling of the POU4F3 protein constructed by the 2xsd protein as the template. (A) Overview of the modeled POU4F3 domain. The protein chain is shown in grey using a ribbon representation, the position of the mutated residue is indicated in green, the DNA is shown as a ball-and-stick model and colored yellow. (B) A close-up view of the mutated residue. The side chains of the wild-type and the mutant residue are shown in green and red, respectively. The figures show that the wild-type Arginine residue interacts with the phosphate backbone of the DNA, while the mutant Lysine residue is slightly shorter than Arginine and cannot make the same interactions with DNA.

We obtained 84.3%−90.6% of the target exome with coverage of at least 10 sequence reads. A total of 153,611variants were discovered in 4 individuals (IV:3, IV:17, V:1 and V:5), and 123,135 variants thereof passed the standard variant quality control (QC). Among the passed variants, 3,395 were shown to be compatible with the phenotype filter (all 3 affected individuals were heterozygous for variants, while the other control individual was homozygous for the reference genotypes) (**Table S1**). Among those 3,395 variants, 21 non-synonymous SNPs were coincided with the region of the linkage peak on 5q31, and 5 of them had an allele frequency of <1% on the variation data from the 1000 Genomes Project and Exome Sequencing Project 6500 (**Table S3**). Among the 5 rare variants, a missense variation Arg326Lys (c.977G>A) was occurring in the *POU4F3* gene (POU domain, class 4, transcription factor 3; MIM# 602460), a previously known gene causing AD-NSHL (DFNA15). The affected Arg326 residue was shown to be evolutionarily conserved across species, and the missense change Arg326Lys was predicted to be deleterious by all 5 algorithms for functional prediction (**Table 2**). On the other hand, 4 missense variants in other genes were predicted to be benign. Sanger sequencing confirmed the WES findings in all 4 individuals (3 affected individuals were heterozygous for Arg326Lys [G/A] and 1 unaffected individual was homozygous for the reference genotype [G/G]). The sequencing analyses targeting Arg326 of *POU4F3* in other family members confirmed that the mutant genotype was running together with the risk haplotype and was completely segregating with the disease phenotype (**Figure 1A**). The molecular modeling analyses demonstrated that the Arg326Lys mutation was predicted to destabilize the 3^rd^ alpha-helix structure of the POU homeodomain of the protein and thereby reduce the proper protein-DNA interactions (**Figures 2A and 2B**). Lastly, the Arg326Lys variant was not observed in a control population aged >60 years with normal hearing (0/100 control chromosomes).

**Table 2:** Five rare missense mutations in the 5q31 linkage region.

Chr	Position(bp, hg19)	Referenceallele	Mutantallele	Reference aa	Mutant aa	Gene	Functional prediction^a^	GERP++RS^b^	29waylogOdds^c^	1000GALL_AF	1000GASN_AF	ESPAA_AF	ESPEA_AF
5	145719967	G	A	R	K	*POU4F3*	+++++	4.62	16.40	0	0	0	0
5	140222014	G	A	A	T	*PCDHA8*	++–––	2.66	12.17	0	0	0	0
5	139189261	A	G	N	S	*PSD2*	–+–––	3.99	9.55	0.0005	0.0017	0	0.0001
5	137548913	C	T	R	Q	*CDC23*	–––––	4.13	9.63	0	0	0	0
5	140557626	G	A	S	N	*PCDHB8*	––U ––	0.10	2.27	0.0014	0.0052	0	0

Abbreviations: Chr, chromosome; aa, amino acid; ALL, all populations; AF, allele frequency; ASN, Asian; AA, African American; EA, European American.

a Functional effects predicted by SIFT, PolyPhen-2, LRT, MutationTaster and MutationAssesor in order. Deleterious effect was denoted as+, neutral effect as –, and unpredicted effect as U.

b Genomic Evolutionary Rate Profiling++ Rejected Substitution score. The larger the score, the more conserved the site.

c Conservation score is calculated based on 29 mammals genomes. The larger the score, the more conserved the site.

## Discussion

The diagnosis of AD-NSHL is typically made based on the phenotype and the inheritance pattern within the family affected, and direct identification of the genetic background is not feasible due to the large number of disease genes/DNFA loci. In this study, we demonstrated that the molecular genetic diagnosis was successful in a large Korean family with DFNA by utilizing a combination approach of linkage analysis using genome-wide SNP markers and WES. The linkage analyses uncovered a 27.1 Mb region on the 5q31 band, which is one of the most gene-rich genomic segments in human, and several known disease loci (genes) have been previously identified on 5q31 in linkage with hereditary HL including DFNA1 (*DIAPH1*), DFNA15 (*POU4F3*), DFNA42, and DFNA54 [17]–[19]. Sequential filtering of the large number of variants obtained from WES pinpointed a missense variant Arg326Lys in *POU4F3* on 5q31, a previously known NSHL gene, as a strong candidate (**Table 2**). The variant was running with the risk haplotype ht-2 reconstructed from SNPs with high LOD scores from linkage analysis (**Figure 1**). Based on the further evidence from Sanger sequencing and bioinformatic analyses, we concluded that Arg326Lys of *POU4F3* on 5q31 was the candidate causative mutation. On molecular modeling analysis, the missense mutation was predicted to result in a deficiency in the proper protein-DNA interaction of the protein by destabilizing the 3^rd^ alpha helix of the POU homeodomain.

The *POU4F3* (POU domain, class 4, transcription factor 3) gene on 5q31 (DFNA15) is one of the two genes belonging to the superfamily of POU domain transcription factors that cause NSHL, along with *POU3F4* on Xq21.1 (DFN3). It was first identified as a causative gene of AD-NSHL in a large Israeli Jewish family by linkage analysis [19]. *POU4F3* was an excellent candidate gene because targeted deletion of both Pou5f3 alleles in mice had been reported to result in complete deafness [20], [21]. Indeed, the POU4F3 protein is a member of the POU family transcription factors and is critical for the maintenance of inner ear hair cells. Following the first report, 4 additional families with NSHL from *POU4F3* mutations have been reported in the literature (**Table 3**) [22]. Collin et al. reported a missense mutation (c.865C>T, p.Leu289Phe) in a large Dutch family by linkage study using short tandem repeat (STR) markers and candidate gene sequencing [23]. They additionally found another family with DFNA15 from a different mutation (c.668T>C, p.Leu223Pro) by screening 30 unrelated families by targeted sequencing of *POU4F3*. The other 2 patients with DFNA15 were Korean families. By screening for DFNA15 in 42 unrelated Korean families with AD-NSHL by sequencing analyses of *POU4F3*, Lee et al. uncovered a family with a frameshift mutation by deletion of 14 nucleotides (c.662_675del14, p.Gly221Glufs*77) [24]. Another Korean family with DFNA15 from a missense mutation in *POU4F3* (c.694G>A, p.Glu232Lys) was reported by targeted massive parallel sequencing of 80 genes [25]. The POU4F3 protein has 2 DNA binding domains, POU-specific domain and POU homeodomain. In particular, the POU homeodomain contains 2 functional nuclear localization signal sequences, monopartite (amino acids 274–278) and bipartite (amino acids 314–331 forming the 3^rd^ alpha helix) [26]. The missense mutations Leu223Pro and Glu232Lys affected the POU-specific domain. Following the previously reported mutation Leu289Phe, Arg326Lys identified in our patient was the 2^nd^ missense mutation in the POU homeodomain. Of note, Leu289Phe was located in the 1^st^ alpha-helix, and Arg326Lys was the first missense mutation affecting the 3^rd^ alpha-helix of the POU homeodomain, which has the bipartite nuclear localization signal. In line with our observations from molecular modeling analyses, a previous bioinformatic study on the missense mutation affecting the Arg329 residue of POU3F4 (Arg329Pro), which corresponds to the Arg325 residue of POU4F3 mutated in our family, demonstrated a detrimental functional consequence of the mutation on the 3^rd^ alpha helix structure [27]. The precise molecular pathophysiological mechanisms would be different between *POU3F4*- and *POU4F3*- related deafness in speculation of the different modes of inheritance (X-linked recessive vs autosomal dominant, respectively) [27]. Collectively, the Arg326Lys mutation of *POU4F3* in our family was predicted to exert its mutational effects by a combination of defects in the stability of its tertiary structure, DNA binding ability, transcriptional activity, and the nuclear localization of the protein. As for the genotype-phenotype correlations in DFNA15, the evidence collected from previously reported 5 families, albeit limited in number, have demonstrated late-onset bilateral progressive hearing loss with significant intra- and interfamilial variabilities in terms of the age of onset and severity of disease and the configuration of audiometric findings (**Table 3**) [19], [23]–[25], [28]. In line with this observation, the family in this report also demonstrated a wide range of onset of hearing loss (early 10 s to late 50 s). The audiograms obtained from 4 affected individuals also showed sensorineural hearing loss with an intrafamilial variability (**Figure 1B**). Further investigation is needed to delineate the precise molecular pathophysiological consequence of this variant in the hearing function.

**Table 3:** Genetics and phenotypes of DFNA15 in previously reported 5 families and in the Korean family in this report.

No.	Ethnicity	Methods	Mutation	Domain	Phenotype	Reference
1	IsraeliJewish	Linkage analysis using STR markersand candidate sequencing	c.884_891del8(p.Ile295Thrfs*5)	NA	18–30 y; progressive; flat todownward-sloping AC	Vahara et al. 1998 (19)
2	Dutch	Linkage analysis using STR markersand candidate sequencing	c.865C>T(p.Leu289Phe)	POU homeodomain(1st alpha-helix)	13 y–20; progressive; flat to gently downward-sloping AC; vestibular hypofunction +/−	Collin et al. 2008 (23), Pauw et al. 2008 (28)
3	Dutch?	Targeted sequencing of *POU4F3*in 30 URF	c.668T>C(p.Leu223Pro)	POU-specific domain	40 y; moderate to severe; affecting mainly high frequencies	Collin et al. 2008 (23)
4	Korean	Targeted sequencing of *POU4F3*in 42 URF	c.662_675del14(p.Gly221Glufs*77)	NA	20 y; severe; downward-sloping AC	Lee et al. 2010 (13)
5	Korean	Targeted massively parallelsequencing of 80 genes andSanger validation in 8 URF	c.694G>A(p.Glu232Lys)	POU-specific domain	27/28 y; moderate/severe; flat/downward-sloping AC	Baek et al. 2012 (25)
6	Korean	Linkage analysis using SNP markersand Sanger validation	c.977G>A(p.Arg326Lys)	POU homeodomain(3rd alpha-helix)	Early 10 s-late 50 s; mild tomoderate flat to gentlydownward-sloping AC	Kim et al. This report

NA, not applicable; y, years of age; AC, audiometric configuration; URF, unrelated families.

Recently, the identification of disease-causing genes and mutations has been accelerated by the unprecedented advances and growing availability of genome research technologies including massively parallel sequencing. However, how to select and determine which variant (s) is (are) the disease-causing mutation (s) in the patient/family under investigation is a big challenge due to the large number of variants obtained from genome or exome sequencing. In order to cope with this challenge, linkage information is critically useful to narrow down the disease locus and thereby to decrease the number of candidate variants, utilizing identity-by-descent information [29]. In the field of hearing genetics, there also have been reports on novel disease genes and mutations by utilizing WES. As in other disease entities, the application of WES is more robust in AR-NSHL (DFNB) especially in consanguineous families with or without homozygosity mapping by SNP-array [30]–[32]. On the other hand, the determination of disease-causing gene/mutation is more challenging in AD-NSHL, and a couple of recent studies reported on successful identification of novel mutations in known DFNA genes [33], [34]. In particular, the study by Park et al. on DFNA20/26 (*ACTG1* mutation) pinpointed the causative mutation from the list of variants obtained from WES utilizing linkage information from SNP markers as in our study [34]. Also in our family, we could decrease the number of functional candidate variants from 444 (genomewide) to 21 by applying the linkage region (5q31) (**Table S3**). Collectively, it is anticipated that WES in combination of linkage information can be successfully utilized in elucidating the genetic background of AD-NSHL. It should also be noted that the technical success and growing availability of genome tests also bring up issues of carrier detection by family study and informed genetic counseling. As in presymptomatic minors carrying the *POU4F3* mutation in our family, for example, a careful approach is mandatory for pre-test and post-test counseling in late-onset diseases such as DFNA [35].

In conclusion, this study demonstrated that WES in combination with linkage analysis using bi-allelic SNP markers successfully identified the candidate locus and mutation in AD-NSHL. The *POU4F3* mutation identified in the Korean family was the first missense mutation affecting the 3^rd^ alpha helix of the POU homeodomain harboring a bipartite nuclear localization signal of the protein. We believe the growing availability of molecular genomic tests opens the possibility of better understanding of molecular mechanisms and genotype-phenotype correlations in NSHL.

## Supporting Information

Table S1 **Family members of the pedigree.**
(DOCX)

Table S2 **Haplotypes in the linkage region on 5q31.**
(DOCX)

Table S3 **Filtering of variants obtained from whole exome sequencing of 3 affected and 1 unaffected individuals.**
(DOCX)
