# Supplementary material for: SNP Linkage Analysis and Whole Exome Sequencing Identify a Novel POU4F3 Mutation in Autosomal Dominant Late-Onset Nonsyndromic Hearing Loss (DFNA15)
Source: PLoS One. 2013 Nov 18;8(11):e79063. doi: 10.1371/journal.pone.0079063 (PMC3832514; doi:10.1371/journal.pone.0079063)
Supplement: Table S1 — Family members of the pedigree. (DOCX) [file pone.0079063.s001.docx]

**Table S1.** Family members of the pedigree

| **No.** | **Age, y/sex** | **Disease status** | **Hearing (age at onset, years)** |
| --- | --- | --- | --- |
| I:2 | Died/F | Patient | Late-onset hearing loss (30s) |
| II:1 | Died/M | Patient | Late-onset hearing loss (40s) |
| III:2 | Died/F | Patient | Late-onset hearing loss (30s) |
| III:5 | Died/M | Patient | Late-onset hearing loss (NA) |
| IV:2 | 68/F | Patient | Late-onset hearing loss (40s) |
| IV:3 | 58/M | Patient | Late-onset hearing loss (40s) |
| IV:4 | 57/F | Normal | Normal |
| IV:9 | 58/M | Patient | Late-onset hearing loss (NA) |
| IV:13 | 56/M | Patient | Late-onset hearing loss (56) |
| IV:16 | 51/F | Patient | Late-onset hearing loss (45) |
| IV:17 | NA/M | Patient | Late-onset hearing loss (30s) |
| IV:20 | 45/F | Patient (proband) | Late-onset hearing loss (21) |
| V:1 | 46/M | Patient | Late-onset hearing loss (NA) |
| V:3 | 43/M | Patient | Late-onset hearing loss (NA) |
| V:4 | NA/F | Normal | Normal |
| V:5 | 40/M | Normal | Normal |
| V:6 | 36/F | Normal | Normal |
| V:8 | 35/F | Normal | Normal |
| V:9 | 33/F | Patient | Late-onset hearing loss (early 10s) |
| V:10 | 27/M | Normal | Normal |
| V:11 | 25/F | Patient | Late-onset hearing loss (early 20s) |
| VI:3 | 4/M | Normal | Normal |
| VI:4 | 15/M | Normal | Normal |
| VI:6 | NA/M | Normal | Normal |
| VI:7 | NA/M | Normal | Normal |

NA, not available.
