# Supplementary material for: SNP Linkage Analysis and Whole Exome Sequencing Identify a Novel POU4F3 Mutation in Autosomal Dominant Late-Onset Nonsyndromic Hearing Loss (DFNA15)
Source: PLoS One. 2013 Nov 18;8(11):e79063. doi: 10.1371/journal.pone.0079063 (PMC3832514; doi:10.1371/journal.pone.0079063)
Supplement: Table S2 — Haplotypes in the linkage region on 5q31. (DOCX) [file pone.0079063.s002.docx]

**Table S2.** Haplotypes in the linkage region on 5q31

| # | Nucleotide sequence^a^ |
| --- | --- |
| 1 | AGATTTGTTTACCTTTTTCGCTGCTC |
| **2**^b^ | **AGATTTGTTTACCTTCTTCATCGCTC** |
| 3 | AGATTTGTTTACCTTCTTCATTACTG |
| 4 | AGGTTTACTTATCTCTTTCATTACTC |
| 5 | AGGTTTACCTATCTCTTTCATTACTG |
| 6 | AGGTTTGTTTACCTCCTTCATTACTG |
| 7 | AGGTTTGCTTACCTTTTTTATTACCC |
| 8 | AGGCTTGCTTACTTCCTACACTGCCG |
| 9 | AGGCCTACTTATCTCTTTCATTACTC |
| 10 | GGACCTACTTATTTCCTTCACCACTG |
| 11 | GGGTTTACTTACCTCCCTCACCACTC |
| 12 | GGGTTTACCTATTTCTTTCATCACTG |
| 13 | GGGTTTGTTTACCTCCTTTACTACTG |
| 14 | GGGTTTGTTTCTCTCTTTCACCACTC |
| 15 | GGGTCCACTTACCCTTCTTACTACCC |
| 16 | GGGCTCACTTCCTTTTCTTACTGCTC |

^a^Nucleotide sequences of 26 SNP loci in the order of rs1541907, rs31202, rs745558, rs1016344, rs248166, rs726847, rs916980, rs1229708, rs10117, rs744675, rs877826, rs2053055, rs801399, rs433623, rs1860991, rs1864003, rs258825, rs33388, rs464727, rs29900, rs248771, rs1480150, rs728937, rs1016256, rs1989154 and rs783779.

^b^The haplotype segregating with the disease phenotype in the family.
