# Supplementary material for: SNP Linkage Analysis and Whole Exome Sequencing Identify a Novel POU4F3 Mutation in Autosomal Dominant Late-Onset Nonsyndromic Hearing Loss (DFNA15)
Source: PLoS One. 2013 Nov 18;8(11):e79063. doi: 10.1371/journal.pone.0079063 (PMC3832514; doi:10.1371/journal.pone.0079063)
Supplement: Table S3 — Filtering of variants obtained from whole exome sequencing of 3 affected and 1 unaffected individuals. (DOCX) [file pone.0079063.s003.docx]

**Table S3.** Filtering of variants obtained from whole exome sequencing of 3 affected and 1 unaffected individuals

| Variant selection | Number of variants |
| --- | --- |
| Total | 153,611 |
| QC standard pass | 123,135 |
| ^a^Compatible with the inheritance pattern | 3,395 |
| ^b^Functionally important | 444 |
| Overlapping with linkage peak on 5q31 | 21 missense variants |
| ^c^Rare in frequency in DB (alternative allele frequency <0.5%) | 5 missense variants^d^ |
| Highly conserved /predicted to be deleterious | Arg326Lys of *POU4F3* |

QC, quality control; DB, database.

^a^Heterozygous in 3 affected individuals and homozygous for the reference nucleotide in 1 unaffected individual.

^b^Variants were annotated to cause or occur at splice site acceptor/donor, start loss, frameshift, stop gain, stop loss, nonsynonymous, codon change, codon insertion, codon change plus codon insertion, codon deletion, and codon change plus codon deletion.

^c^Both in 1000 Genomes and ESP6500 or <1% in 1000 Genomes ASN (Asian).

^d^See Table 2 in the main text.
